# Do we need race-specific resting metabolic rate prediction equations?

**DOI:** 10.1038/s41387-019-0087-8

**Published:** 2019-07-29

**Authors:** James Reneau, Brittaney Obi, Andrea Moosreiner, Srividya Kidambi

**Affiliations:** Division of Endocrinology and Molecular Medicine, Department of Medicine, Medical College of Wisconsin, 8701 Watertown Plank Road, Milwaukee, WI USA

**Keywords:** Obesity, Obesity

## Abstract

**Background:**

Resting metabolic rate (RMR) is a key determinant of daily caloric needs. Respirometry, a form of indirect calorimetry (IC), is considered one of the most accurate methods to measure RMR in clinical and research settings. It is impractical to measure RMR by IC in routine clinical practice; therefore, several formulas are used to predict RMR. In this study, we sought to determine the accuracy of these formulas in determining RMR and assess additional factors that may determine RMR.

**Methods:**

We measured RMR in 114 subjects (67% female, 30% African American [AA]) using IC. Along with standard anthropometrics, dual-energy X-ray absorptiometry was used to obtain fat-free mass(FFM) and total fat mass. Measured RMR (mRMR) by respirometry was compared with predicted RMR (pRMR) generated by Mifflin–St.Joer, Cunningham, and Harris–Benedict (HB) equations. Linear regression models were used to determine factors affecting mRMR.

**Results:**

Mean age, BMI, and mRMR of subjects were 46 ± 16 years (mean ± SD), 35 ± 10 kg/m^2^, and 1658 ± 391 kcal/day, respectively. After adjusting for age, gender, and anthropometrics, the two largest predictors of mRMR were race (*p* < 0.0001) and FFM (*p* < 0.0001). For every kg increase in FFM, RMR increased by 28 kcal/day (*p* < 0.0001). AA race was associated with 144 kcal/day (*p* < 0.0001) decrease in mRMR. The impact of race on mRMR was mitigated by adding in truncal FFM to the model. When using only clinically measured variables to predict mRMR, we found race, hip circumference, age, gender, and weight to be significant predictors of mRMR (*p* < 0.005). Mifflin–St.Joer and HB equations that use just age, gender, height, and weight overestimated kcal expenditure in AA by 138 ± 148 and 242 ± 164 (*p* < 0.0001), respectively.

**Conclusion:**

We found that formulas utilizing height, weight, gender, and age systematically overestimate mRMR and hence predict higher calorie needs among AA. The lower mRMR in AA could be related to truncal fat-free mass representing the activity of metabolically active intraabdominal organs.

## Introduction

Obesity is a serious global health concern due to its association with metabolic and cardiovascular diseases^1^. Weight loss can decrease health risks associated with obesity^2,3^. Primary strategy for weight loss is caloric restriction with addition of pharmaceuticals and/or bariatric surgery when necessary and exercise therapy for weight maintenance^4–7^. Caloric restriction relies on consumption of fewer calories than the total daily energy expenditure (EE) resulting in mobilization of energy stored in fat and subsequent weight loss^8^. Guidelines recommend that caloric restriction be individualized and is prescribed after evaluating daily EE^3,9–11^, which is a function of resting metabolic rate (RMR), thermic effect of food, non-exercise activity thermogenesis, and exercise^12–14^. Of all, RMR is the major contributor to daily EE constituting 60–80% of its value and varies based on the activity level of the individual^15,16^.

RMR, also referred to as resting EE, is typically defined as the amount of energy expended when an individual is awake and is in a post-absorptive and a thermoneutral state^17–19^. A reliable method to measure RMR (mRMR) is respirometry, a form of indirect calorimetry^20^, which involves measurement of oxygen consumed (VO2) and carbon dioxide emitted (VCO2), and requires use of expensive specialized equipment (i.e., metabolic cart)^21^, extra clinic time for proper measurement, and a properly trained staff^22^. This is not a commonly available method and is seldom used in routine clinical practice. It is, therefore, common to use prediction equations to derive predicted RMR value (pRMR)^9,10^. These equations typically use age, gender, height, and weight to derive the RMR. However, there is a significant debate over the validity of these equations, as they may over- or underestimate caloric needs since the equations are currently being used in significantly different kinds of populations and conditions than they were originally created for^23–25^. Most commonly used equations (e.g., Harris–Benedict and Mifflin–St.Joer) used predominantly Caucasian populations to develop and validate equations^9,10^. Studies suggest that additional factors such as body composition, ethnicity, medications, and ambient temperature may play a role in the determination of RMR^17,26^.

The purpose of this study was to test the accuracy of commonly utilized RMR formulas (Mifflin-St.Joer, Cunningham, and Harris–Benedict) in predicting RMR when compared with RMR measured by respirometry in a mixed population and to evaluate the influence of additional clinical measurements on mRMR.

## Methods

### Subjects

One-hundred and fourteen subjects were recruited from Southeastern Wisconsin area. We pooled subjects recruited for two different studies to increase the sample size and included subjects who had RMRs measured by respirometry. The first study included only African American (AA) subjects between ages 18 and 45 years to evaluate the effect of adiposity on the vascular function. The second study included subjects of all races over the age of 18 years, although almost all the subjects were Caucasian. The purpose of the second study was to evaluate characteristics of central and peripheral adiposity. Exclusion criteria for both studies included the following: pregnancy, nursing, and active malignancy. All subjects provided informed consent and protocols were approved by the Froedtert Hospital and the Medical College of Wisconsin Institutional Review Board.

### Anthropomorphic measures

Phenotypic measures such as height, weight, waist circumference, and hip circumference were measured by the same bionutritionist for both studies to ensure reproducibility using well-calibrated equipment: (1) a scale (Scale-tronix 5200, Welch Allen, Skaneateles Falls, NY, USA); (2) a fixed wall-scale stadiometer (Harpenden stadiometer 602VR, Holtain, Wales, UK); and (3) a tape measure (Gulick II, Country Technology, Inc., Gays Mills, WI). Body mass index (BMI) was calculated using the standard formula. Weight and height of all subjects were measured in scrubs and without shoes. Waist circumference measurements were taken at the level of umbilicus. Hip circumference measurements were taken at the level of widest part of buttocks. An average of three measurements was used for each phenotype.

### Dual energy X-ray absorptiometry (DXA)

Dual-energy X-ray absorptiometry (DXA) measurements were performed using a total body scanner (iDXA by General Electric Lunar Medical Systems, Madison, WI, USA). Quality assurance block phantom and tub phantom spine scans were completed each morning before subjects were scanned. Cross-calibration procedures were followed before and after hardware changes or updates. Quality assurance reports are reviewed and monitored for accuracy and precision by a trained operator. A series of transverse scans from head to toe were performed by a trained operator. Algorithms used for analysis were provided by the software program as part of the iDXA, which allows delineation of different regions of interest. Total percent body fat, total fat mass, total fat-free mass, regional total fat mass, and regional fat-free mass were measured.

### Resting metabolic rate

Respirometry (Metabolic cart, Parvo TrueOne2400, Sandy, UT, USA) was used to obtain mRMR. The metabolic cart measures oxygen consumed (VO2) and carbon dioxide produced (VCO2) by participants and calculates the EE using the modified Weir equation (EE = [3.941 × VO2] + [1.106 × VCO2])^27^. All participants were tested after a 10 h overnight fast while lying supine and in a relaxed condition. Efforts were made to achieve thermoneutral conditions by altering the ambient temperature based on reports from subjects being warm or cold. All participants were asked to refrain from physical activity for 24 hours prior to the measurements. Prior to each test, flowmeter and gas analyzer calibrations were completed using diluted gas of 16% oxygen and 1% carbon dioxide, and participants were fitted for the canopy interface. Once participants were comfortable and acclimated to the equipment, measurements were taken for 20 min and included ventilatory rate, oxygen consumption (VO2), carbon dioxide production (VCO2), and respiratory exchange ratio (RER).

### Prediction equations

Predictive equations used to calculate pRMR in this study are shown in Table 1. Of the three equations, the Cunningham’s formula’s variable input uses only lean body mass derived from a prediction equation^28^. In our study, we used fat-free mass measured by DXA to represent lean body mass. The Mifflin–St.Joer prediction equation uses height, weight, age, and gender^10^, whereas Harris–Benedict equation uses gender-specific formulas with height, weight, and age^9^.

**Table 1:** Commonly used prediction equations

Mifflin–St.Joer^10^	RMR = 9.99 (weight) + 6.25 (height) − 4.92 (age) + 166 (sex) − 161 [sex: men, 1; women, 0]
Cunningham^28^	RMR = 500 + 22 (LBM)
Harris–Benedict^9^	Men: RMR = 13.75 (weight) + 5 (height) − 6.76 (age) + 66.47 Women: RMR 9.56 (weight) + 1.85 (height) − 4.96 (age) + 655.1

*LBM* lean body mass, *RMR* resting metabolic rate

### Statistical analysis

Descriptive statistics were expressed as mean ± SD. AA and Caucasian groups were compared using *χ*^2^- and Wilcoxon’s rank-sum tests for categorical and continuous variables, respectively. Predicted RMRs were compared with mRMRs using a paired *t*-tests. Pearson’s correlation coefficients were used to evaluate univariate relationship between patient characteristics and mRMR. Multivariate linear regression models were constructed using a step-wise selection process to obtain variables that offer the best predictive value with mRMR as the dependent variable. Entrance into models were determined by an *α*-value < 0.05. The first model used all subject characteristics that were measured and included age, gender, weight, height, race, BMI, waist and hip circumferences, waist-to-hip ratio, total fat mass, fat-free mass, and RER. Another linear regression model was generated using variables that are measurable in the clinic without specialized equipment and included age, gender, weight, height, race, waist and hip circumferences, and waist-to-hip ratio. Two separate linear regressions models were generated by picking variables to evaluate the role of regional fat-free mass on mRMR instead of using a step-wise selection process. A *p*-value of <0.005 was considered significant to account for multiple comparisons. Statistical analyses were performed with SAS software (version 9.1; SAS Institute, Inc., Cary, NC, USA).

## Results

A total of 114 subjects (67% women, 30% AA) were included in the analyses. Subject characteristics are shown in Table 2. AA subjects were younger, had lower waist circumference, waist-to-hip ratio, and total fat mass compared to Caucasian subjects. Table 3 shows regional adiposity characteristics derived from DXA. AA subjects had a lower percentage of total body and upper body fat (arm and truncal regions).

**Table 2:** Subject characteristics (mean ± SD)

Variable	All (*n* = 114)	Caucasian (*n* = 80)	African American (*n* = 34)
Female (%)	78 (67)	60 (74)	18 (51)*
Age (years)	46 ± 16	51 ± 15	35 ± 11**
BMI (kg/m^2^)	35 ± 10	36 ± 11	33 ± 9
Height (cm)	168 ± 9	168 ± 11	169 ± 6
Weight (kg)	98 ± 29	101 ± 31	93 ± 25
Waist circumference (cm)	109 ± 24	113 ± 24	101 ± 21*
Hip circumference (cm)	120 ± 21	123 ± 22	114 ± 16
Waist-to-hip ratio	0.91 ± 0.09	0.93 ± 0.1	0.88 ± 0.08*
Total fat mass (kg)	41.3 ± 21	44.3 ± 21	34 ± 20*
Fat-free mass (kg)	57 ± 12.4	56 ± 13	58 ± 10
Total fat percent (%)	41 ± 11	44 ± 10	36 ± 12*

*BMI* body mass index

*p-value comparing Caucasians and African Americans

*p < 0.05; **p < 0.0001

**Table 3:** Regional adiposity distribution (mean ± SD)

Characteristic	All subjects (*n* = 113)	Caucasian (*n* = 79)	African American (*n* = 34)
Total fat mass (kg)	98 ± 29	100 ± 30	93 ± 25
Trunk	49 ± 18	51 ± 20	44 ± 15*
Arms	10 ± 2	10 ± 3	10 ± 2
Legs	31 ± 8	31 ± 9	31 ± 8
Percent fat mass (%)	41 ± 11	54 ± 10	36 ± 12**
Trunk	44 ± 13	47 ± 12	39 ± 13**
Arms	39 ± 12	42 ± 11	32 ± 13***
Legs	39 ± 11	41 ± 10	36 ± 11*
Fat-free mass (kg)	57 ± 12	56 ± 13	58 ± 10
Trunk	26 ± 6	27 ± 7	26 ± 5
Arms	6.4 ± 2	6 ± 2	7.3 ± 2***
Legs	20 ± 5	19 ± 5	21 ± 4

*p-value comparing Caucasians and African Americans

*p < 0.05, **p < 0.01, ***p < 0.001 (*p*-values < 0.005 was considered significant to account for multiple comparisons)

### Resting metabolic rate: predicted vs. measured

**Fig. 1:**
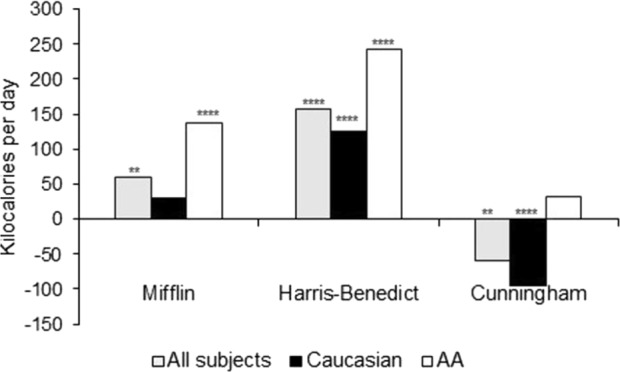
Differences between predicted RMR and measured RMR using prediction equations. Figure showing differences between predicted RMR (by Mifflin–St.Joer, Harris–Benedict, and Cunningham equations) and measured RMR (by respirometry) in all subjects, Caucasians, and AA subjects. AA: African American; RMR: resting metabolic rate. **p < 0.005, ****p < 0.0001

The mean mRMR for the overall cohort was 1658 ± 391 kcal/day (Table 4). Figure 1 shows average differences between mRMR and pRMR using various equations. The Mifflin–St.Joer equation significantly overestimated the mRMR value among AA (*p* < 0.0001) but not for Caucasians after adjustment of multiple comparisons. Cunningham equation underestimated caloric requirements for Caucasians, whereas no significant difference was noted in AA. Harris–Benedict equation overestimated significantly caloric expenditure significantly in both groups (Supplementary Table 1).

**Table 4:** Resting metabolic rate: measured and predicted

Variable	All	Caucasian	African American
Measured RMR	1658 ± 391	1672 ± 408	1621 ± 346
Respiratory exchange ratio	0.83 ± 0.06	0.82 ± 0.06	0.85 ± 0.07*
Predicted RMR			
Mifflin–St.Joer	1718 ± 354	1704 ± 374	1759 ± 297
Cunningham	1598 ± 274	1578 ± 288	1654 ± 230
Harris–Benedict	1816 ± 394	1798 ± 402	1864 ± 375

*RMR* resting metabolic rate

*p-value comparing Caucasians and African Americans

*p < 0.05.

### Relationship between RMR and other anthropometric measures

In the univariate analyses, mRMR correlated positively with weight, BMI, waist and hip circumferences, and total fat and fat-free mass in both Caucasians and AA (Table 5). Multivariate linear regression model was generated using a step-wise selection process with all available measurements. The model consisted of age, gender, race, height, weight, waist and hip circumferences, waist-to-hip ratio, total fat mass, fat-free mass, and smoking status. The model generated was significant (*p* < 0.0001) with an *R*^2^-value of 0.88. Among all variables in the model, statistically significant positive predictors were total body fat mass and fat-free mass (*p* < 0.0001). For every 1 kg increase in total body fat mass, mRMR was increased by 5.9 kcal/day. For every 1 kg increase in fat-free mass, mRMR was predicted to increase by 28.2 kcal/day. Significant negative predictors were age and AA race (*p* < 0.005). As age increased by 1 year, mRMR decreased by 4.3 kcal/day. The AA race was associated with a 144 kcal/day decrease in mRMR. To evaluate whether regional distribution of fat-free mass affects mRMR, we generated a regression model utilizing fat-free mass in each region. The race effect in the above model was completely mitigated when total fat-free mass was substituted for truncal fat-free mass. After adjusting for age and total body fat mass, fat-free mass in the upper body (arms and trunk) was associated positively with mRMR in Caucasians (*p* < 0.005), while the same regions had an insignificant impact on mRMR in AA. However, fat-free mass in the lower body (legs) was positively associated with mRMR in AA (*p* < 0.005).

**Table 5:** Correlations between RMR and anthropometric measures

Parameter	All subjects	Caucasian	African American
Age	−0.01	−0.17	−0.03
Weight	0.85**	0.86**	0.79**
Height	0.45**	0.48**	0.40
BMI	0.69**	0.70**	0.68**
Waist circumference	0.71**	0.73**	0.68**
Hip circumference	0.63**	0.64**	0.60*
Waist-to-hip ratio	0.45**	0.43*	0.53*
Total fat mass	0.66**	0.69**	0.58*
Fat-free mass	0.87**	0.89**	0.86**

*BMI* body mass index

*RMR* resting metabolic rate

*p < 0.005, **p < 0.0001

The results of the multiple linear regression model generated using only clinically measured variables (age, sex, height, weight, BMI, waist and hip circumferences, and smoking history) was significant with an *R*^2^-value of 0.83 (*p*-value < 0.0001). The only significant positive predictor of mRMR was weight, which was a 15.4 kcal/kg/day increase in RMR for every kg increase in weight. The significant negative predictors were age, hip circumference, female sex, and AA race. With every year increase in age, RMR was predicted to decrease by 5.3 kcals/day. For every centimeter increase in hip circumference, RMR was estimated to decrease by 6.7 kcals/day. Female sex was associated with a decrease in RMR of 127 kcals/day compared with male sex. The AA race was associated with a 158 kcal/day decrease in mRMR compared with Caucasians. The negative relationship between hip circumference and mRMR was unexpected and appears to be a surrogate marker for total body fat percent (*r* = 0.8, *p* < 0.0001). As fat mass has lower mRMR compared with fat-free mass, individuals with higher hip circumference are expected to have lower mRMR, capturing that effect. Substituting total body fat percent into our model using clinical variables negated the effect of hip circumference confirming the mediation effect of total body fat percent (data not shown).

## Discussion

In the current study conducted in two cohorts of AA and Caucasian populations, we found that the AA race is the most significant negative predictor of mRMR after adjusting for age, sex, BMI, total fat mass, and fat-free mass. Consequently, commonly used RMR prediction equations (e.g., Mifflin–St.Joer) utilizing height, weight, age, and gender systematically overestimated daily caloric requirements in AA. However, race effect was completely mitigated after adjustment for truncal fat-free mass. These findings highlight the role of race and regional body composition in determining mRMR and if ignored may overestimate daily EE in AA.

**Fig. 2:**
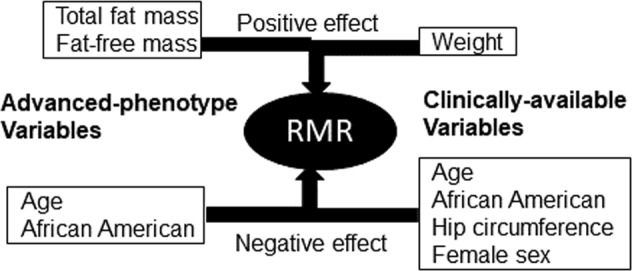
Determinants of resting metabolic rate. Demographic and anthropometric variables with positive or negative influence on resting metabolic rate in multivariate regression analyses

It is well-known that RMR is affected by several inherent factors including age, sex, body weight, and body composition (Fig. 2). Our study findings are in line with previous studies that showed that mRMR correlates positively with BMI, total fat mass, and fat-free mass^29,30^. Regression analyses revealed that after adjustment for age, sex, body weight, and height, RMR was determined positively by total fat mass and fat-free mass. AA race was the most significant negative predictor of mRMR even after adjustment for total fat mass and fat-free mass. Although similar findings of AA race effect on RMR have been reported by previous studies^31–33^, race is not considered in formulas used to determine caloric requirements in the clinical practice. Respirometry is one of the most-reliable methods to mRMR in a clinical setting, which however is not widely available for use in clinical practice, although some hand-held devices are currently available^34^.

In lieu of respirometry, various prediction equations are used to derive pRMR for assessment of daily caloric requirements in clinic^9,10,28^. Due to lower mRMR in AA, equations that utilize just height, weight, sex, and age (e.g., Mifflin–St.Joer and Harris–Benedict) to predict mRMR performed dismally among AA in our cohort by significantly overpredicting RMR. For the same height, weight, gender, and age characteristics as Caucasian subjects, mRMR in AA was 138 kcal less per day (*p* < 0.0001) compared with what was predicted by Mifflin–St.Joer equation, which is used extensively in clinical practice irrespective of the race of the patient. The difference among Caucasians was insignificant between mRMR and RMR value predicted by Mifflin–St.Joer equation, which is probably expected, as this formula was derived based on Caucasian population in the 1990s. Utility of Harris–Benedict equation has also been questioned with its inaccuracies in obese and racial minority populations^35^. In our cohort, it was the worst performer of the three equations tested in both AA and Caucasians with almost 10% overestimation in the overall sample (and 18% in AA). The Cunningham equation, which uses fat-free mass to estimate RMR, was better than the above two in predicting the RMR in AA but significantly underestimated mRMR in Caucasians. Despite large amounts of data indicating that prediction models of RMR that do not take into consideration either race or fat-free mass overestimate daily caloric requirements among AA, formulas such as Mifflin–St.Joer continue to be used in routine clinical practice.

Multiple factors have been attributed to lower resting EE in AA and include higher fat mass, lower fat-free mass, lower fitness rates, lower sleep duration, and differences in uncoupling protein genes among AA^36–40^. Several studies, including ours, have shown a favorable body composition profiles including higher fat-free mass among AA. However, the lower RMR in AA persisted in our study even after adjustment of total fat mass and fat-free mass along with age, gender, and BMI. One previous study attributed this lower RMR to smaller organ sizes in AA, which are measured as fat-free mass^41^. Investigators used magnetic resonance imaging (MRI) to measure the sizes of multiple organs with high metabolic rates including the liver, kidney, brain, spleen, and heart in 42 men and women. They found that racial differences in RMR were no longer significant once lean mass with organ size was considered. They concluded that AA have smaller sized organs with high baseline energy consumption (e.g., liver, kidney, etc.) and therefore expend less energy in a resting state^41^. Mean BMI of subjects in this study was <30 kg/m^2^ and therefore may not be representative of populations struggling with obesity. In another study by Hunter et al.^42^, non-Hispanic White women had a larger metabolically active organs within the truncal region compared with AA women and speculated that this may account for some of the variance seen in total and resting EE between the ethnic groups^42^.

Based on the above studies, we assessed the impact of regional distribution of fat-free mass as a surrogate to capture potential effect of high energy-consuming abdominal organs, on RMR. We found that when using fat-free mass in the truncal region in the place of overall fat-free mass, the effect of race was rendered nonsignificant. Furthermore, we found that fat-free mass in the truncal region contributed to a statistically significant increase in mRMR in Caucasians, while there was no such increase in AA again, indicating low contribution of abdominal fat-free mass to RMR overall in AA. This is the first study to show the impact of truncal fat-free mass on mRMR in AA and it is plausible that organ size was captured by using truncal fat-free mass measured by DXA scan. Measurement of specific organ sizes is cumbersome using MRI and development of a surrogate measure would be helpful to further investigate this phenomenon in clinical studies. Although a direct comparison of organ sizes with truncal fat-free mass needs to be carried out, if DXA represents an alternative to MRI-based measurements, it could serve as a surrogate marker for organ size in clinical studies. Interestingly, Jones et al.^39^ found that the DXA measured fat-free mass contained more skeletal muscle in AA women than it did in Caucasian women, indicative again of smaller metabolically active organ sizes/weights in AA.

Lastly, regression models utilizing only clinically measurable variables indicated female sex and AA race were associated with significantly lower RMR even after adjustment of age, height, weight, and waist circumference, clearly indicating that currently used prediction formulas are inadequate to estimate calorie requirements. Interestingly, hip circumference was associated with slight decrease in RMR, perhaps by acting as a surrogate marker for higher total fat mass compared with fat-free mass. Larger studies are needed to determine whethe raddition of hip circumference as a surrogate for fat mass to prediction formulas would improve prediction of mRMR. Luhrmann et al.^43^ recommended applying waist-to-hip ratio to improve clinical accuracy of predictive equations in an elderly German population.

Obesity in the United States disproportionately affects AA, particularly women, and etiology has been considered multifactoria^44^. Lower RMR has been hypothesized to be a contributor to increase in obesity prevalence in AA^33,45^. AA women also have been shown to lose less weight despite similar adherence to interventions and it was attributed to lower energy requirements^46,47^. It has been previously shown that lower RMR predicted future weight gain in Pima Indians and pregnant AA women^12,48^. Findings of lower RMR in AA in this study and others warrant further exploration into the mechanisms of lower RMR, its contributions to prevalence of obesity in AA, and need for race-specific RMR prediction equations.

Limitations of our study include lack of data on diet, physical activity, sleep, and information at the molecular levels, which have been thought to account for some of the discrepancies in pRMR^37^. In addition, we have to acknowledge that respirometry is not the gold standard method to measure RMR, as it does not provide a comprehensive measure of all metabolic processes that occur in vivo; however, it is the most commonly employed method to obtain RMR reliably^49,50^. Moreover, we used modified Weir equation in order to derive RMR from the respirometry data, which does not consider oxidation of substrates other than carbohydrates, proteins, and fats, and may result in some discrepancies in the calculation of RMR based on their diet. Our study sample size is small, has more women than men, and are more obese than normal-weight subjects. We also acknowledge that our AA subjects are much younger than our Caucasian subjects, although we believe that this should not affect the results of the study, as age is taken into consideration in these equations. However, much of the analysis conducted with BMI as continuous variable and having a range of BMIs is a strength of our study. Larger studies are needed to see whether there is a sex difference in the discrepancy in RMR, as some studies seem to indicate the racial differences may be limited to women^33^. Lastly, this was a cross-sectional study and therefore we cannot attribute the rising prevalence of obesity in AA women to lower RMR.

Apart from the implication toward etiology of obesity, lower mRMR has practical ramifications in day-to-day clinical practice in determining daily caloric requirements by the dieticians in individuals who are attempting to lose weight. As the current validated formulas are flawed, some have suggested use of race-specific RMR formulas to improve accuracy of predicative equations^25,51^, but others have concluded that this discrepancy in RMR in AA is not clinically relevant^47,52^. Our study found that AA have a lower daily EE by an average 144 kcal/day compared with Caucasian participants of the same height, weight, age, and gender, and after adjustment for total fat mass and fat-free mass. Even a difference of 100 kcal/day in RMR, while appearing insignificant, could contribute to 10 lb weight gain per year in AA when all other factors are comparable to Caucasians. Racial differences were completely mitigated after adjustment for truncal fat-free mass, indicating potential role of smaller metabolically active organ sizes in AA in determining RMR. It seems imperative that these racial differences should be taken into consideration and formulas containing a race factor or regional fat-free mass are needed to accurately predict RMR in AA.

Data availability: All data will be provided in excel spreadsheet without restriction upon request.

Authors contributions: S.K. conceived the study, obtained funding, supervised data acquisition, analyses, interpreted the results, and edited the draft manuscript. B.O. and A.M. assisted in data acquisition. J.R. performed data analyses and drafted the manuscript.

## Supplementary information

Supplemental table 1: Differences between measured and predicted RMRs using various equations
