# Supplementary material for: Do we need race-specific resting metabolic rate prediction equations?
Source: Nutr Diabetes. 2019 Jul 29;9:21. doi: 10.1038/s41387-019-0087-8 (PMC6662665; doi:10.1038/s41387-019-0087-8)
Supplement: Supplementary file 1 — Supplemental table 1: Differences between measured and predicted RMRs using various equations [file 41387_2019_87_MOESM1_ESM.docx]

Supplemental table 1: Differences between measured and predicted RMRs using various equations

|  | Mifflin | Cunningham | Harris-Benedict |
| --- | --- | --- | --- |
| All Subjects | 60 ± 183^*^ | -60 ± 205^*^ | 158 ± 204^****^ |
| Caucasian | 31 ± 187 | -95 ± 198^****^ | 126 ± 210^****^ |
| African American | 138 ± 148^****^ | 33 ± 195 | 242 ± 164^****^ |

RMR: resting metabolic rate

^*^p-value comparing measured RMR with predicted RMR by each equation

^*^p<0.05; ^****^ p< 0.0001
